# Cannabidiol and refractory epilepsy: parental and caregiver perspectives of participation in a compassionate access scheme

**DOI:** 10.1186/s12913-022-07592-4

**Published:** 2022-02-10

**Authors:** S. Harte, Y. Singh, S. Malone, H. Heussler, G. Wallace

**Affiliations:** 1 The University of Queensland, School of Medicine, Brisbane, Australia; 2 Queensland Children’s Hospital, South Brisbane, Australia

**Keywords:** Refractory epilepsy, Cannabidiol, Expectations, Caregiver, Clinical trial, Experimental

## Abstract

**Background:**

The *Compassionate Access Scheme* (CAS) being delivered through the *Queensland Children’s Hospital* is designed to allow access to an investigational purified Cannabidiol oral solution to paediatric patients with severe refractory epilepsy. The objectives of this study were to conduct semi-structured interviews to:

1. Understand families’ expectations and attitudes about the use of an investigational cannabinoid product for their child’s seizures;

2. Understand families’ perceptions of Cannabidiol’s efficacy for their child’s seizures; and other aspects of their child’s behaviour, quality of life and/or cognition.

**Methods:**

Children aged 2-18 years had been enrolled in, or were enrolled in a compassionate access scheme for Cannabidiol at the time of the study. Semi-structured interviews (*n* = 19) with parents or caregivers (*n* = 23) of children diagnosed with refractory epilepsy were voice-recorded, transcribed and analysed to generate common themes.

**Results:**

Key themes emerged relating to seizure activity, family and school engagement, drug safety and legal access, efficacy, clinical support, social acceptance of the medication and program delivery. The use of Cannabidiol was perceived to have benefits in relation to reducing the severity and frequency of seizure activity for almost a third of patients experiencing refractory epilepsy. Participants described other benefits including improved social engagement, wakefulness and a reduction of side effects related to a reduction of conventional medication dosage.

**Conclusion:**

This study provided unique perspectives of families’ experiences managing untreatable epilepsy, their experiences with conventional and experimental pharmacological treatments and health services. Whilst families’ perceptions showed the use of Cannabidiol did not provide a therapeutic reduction in the seizure activity for all patients diagnosed with refractory epilepsy, it’s use as an additional pharmacological agent was perceived to provide other benefits by some patient families.

## Background

Epilepsy is commonly defined as a disorder of the brain characterised by an enduring predisposition to generate epileptic seizures with unknown origin or, as a result of infection, injury, tumour or disease [1, 2]. Global prevalence of epilepsy has been estimated at 45.9 million people in 2016 [2].

Refractory, or untreatable epilepsy has been defined as the “diagnosis of drug-resistant epilepsy as a result of a failure of adequate trials of two tolerated and appropriately chosen and used anti-epileptic drug (AED) schedules (whether as monotherapies or in combination) to achieve sustained seizure freedom” [3]. Untreatable or refractory epilepsy has been associated with increased risk of mortality and morbidity including sudden unexplained death, neuropsychological impairment, psychiatric and behavioural disturbances and psychosocial challenges [4–6].

The burden of epilepsy disease has been calculated in disability adjusted life years (DALYs), a population measure of health loss accounting for years of life lost (YLL) and years of life lived with disability (YLD) [2]. Whilst the burden of disease has been calculated for idiopathic presentation of epilepsy as approximately 0.5% of all DALYs for all disease, the burden of disease for untreatable epilepsy is estimated to be even higher, approximately sevenfold [7].

The treatment and results associated with medicinal cannabis in the form of Cannabidiol (CBD) for refractory epilepsy associated with Dravet and Lennox-Gastaut Syndromes has prompted formal International discussion with published clinical trial results, conference presentations and Government position statements, as well as informal anecdotal accounts shared in social media groups [8–11]. Over time, increasing interest in CBD led families to request the legal provision of CBD as an option in situations where other treatment modalities including pharmacological, dietary, surgical, physical and behavioural therapies have been unsuccessful [12]. The *Compassionate Access Scheme* (CAS) being delivered through the Queensland Children’s Hospital is designed to allow access to an investigational purified CBD oral solution to paediatric patients with severe refractory epilepsy, and similar to other access schemes through Australia and the world [9, 12].

This study aimed to conduct semi-structured interviews to understand families’ expectations and attitudes about the use of an investigational cannabinoid product for their child’s seizures. Furthermore, the interviews were intended to gain an understanding of families’ perceptions of the efficacy of CBD for their child’s seizures; and other aspects of their child’s behaviour, quality of life and/or cognition.

## Methods

A qualitative approach was undertaken to develop a semi-structured interview format for the study.

### Setting

The Centre for Clinical Trials in Rare Neurodevelopmental Disorders (CCTRND) was established to conduct clinical trials for patients attending the Queensland Children’s Hospital (QCH) Neurology and Child Development clinics. The supply of Epidiolex™ as an oral preparation was made available for children experiencing severe refractory epilepsy. The scheme allowed for 40 eligible patients to participate at any time.

For clinicians, the CCTRND provided usual medical and allied health services for patients attending clinical appointments. Clinical trial staff were recruited, trained and accredited according to Good Clinical Practice (GCP) standards accepted by International regulatory bodies and pharmaceutical compliance officers operating in Australia and other countries to administer the CAS program [13]. Patient clinical and trial records were maintained according to Hospital and Health Service and Clinical Trial requirements, ethical and governance compliance standards. The medications being trialled were supplied through direct partnership with an approved pharmaceutical company.

### The study

The study was conducted as a thematic analysis of semi-structured interviews with parents or caregivers (*n* = 23) of children aged 4-18 years, diagnosed with refractory epilepsy of varied causes. A diagnosis of refractory epilepsy was based on the failure of at least two treatments, which could include medication and other therapies such as ketogenic diet and vagal nerve stimulation. Children had been enrolled in or were currently enrolled in the compassionate access scheme (CAS) for CBD at the Queensland Children’s Hospital in South Brisbane, Queensland. Participants in the study had been screened for eligibility for the CAS program and supported by their usual Neurology team clinicians. Through the scheme, all patients therefore had legal, approved access to an experimental drug treatment according to regulated Pharmacy conditions. Data was recorded to monitor and evaluate aspects of the drug treatment in an open-label trial design. All participants in the cannabidiol trial were contacted by telephone by the first author and were given an explanation of the study. Interested participants were provided with a participant information sheet via email before participating in the study and were given an opportunity to ask questions prior to the researcher obtaining written consent. One family declined to participate in the study and other families did not respond to the invitation to participate. Recruitment was discontinued once all interested participants had been interviewed.

The interview questions were adapted for the interviews from a survey format questionnaire that was made available through the Epilepsy Action Australia organisation [14]. The original survey format was delivered online with approximately two thirds of questions presented as dichotomous yes/no or multi choice options and one third free text questions. For this study, 36 questions were used to guide the interview. Questions were asked to gather background information about children’s seizure history before and during the trial, reasons for joining the trial, expectations in relation to cannabidiol medication, perceived effect of cannabidiol medication for seizures or other effects, social factors an opportunity to provide unstructured responses. The other main difference between the current study, and the original study format was that the online questionnaire was available to any person diagnosed with epilepsy or who knew someone diagnosed with epilepsy, whereas this study recruited parents and caregivers of children who had participated in the CAS program.

### Data collection

Data included 19 interviews collected in 2019 at the research centre by the first author, a trained qualitative researcher, employed as a senior research officer for the study. Parents and caregivers were given the option of being interviewed individually or together. Four of the interviews were with two parents or caregivers present. Participation in the trial varied for the participants as some families were enrolled in the CAS program at the time of interview and some had completed the trial or had withdrawn from the trial. Data was collected using a voice-recording device. As the interview questions for this study were delivered face to face or over the phone, mostly as open-ended questions, participants were able to provide unstructured answers. Interview duration ranged from 25 to 75 min. There were few dichotomous questions where yes or no was the only possible answer. Data collection included recording, de-identifying and transcribing the interviews into text. Field notes were written after the interviews.

### Data analysis

Data coding and correlation was performed with the first author and consulting CAS program Neurologist using clinical file notes to confirm patient diagnoses, CBD dosing information and clinical efficacy. Data analysis then included a thematic analysis of the transcriptions to generate common trends. Utilising inductive and deductive methods, themes were identified and grouped until no new themes emerged and saturation was deemed to have been achieved. Themes and extracted quotes have been provided here to illustrate the findings. The thematic findings were discussed with one participant for agreement in a member-checking interview [15].

## Results

Parents and caregivers of children in the CAS program described their expectations for their children’s involvement in the CBD trial on a background of their experiences managing their children’s chronic health condition. Participants outlined implications for managing uncontrolled seizure activity, status epilepticus, unsuccessful pharmacological, dietary and medical interventions and the impact on family, social and economic participation. Parents and caregivers reported that CBD treatment had benefits in relation to reducing the severity and frequency of seizure activity for some of the children, but not all experiencing refractory epilepsy. At the time of the study, 2 participants were weaning off CBD and 5 of the families (37%) had withdrawn from the CAS program. Reasons for withdrawal that were provided were the lack of improvement in seizure activity for their child and side effects that included daytime sedative effect with nocturnal insomnia and increased dribbling. For other patients, benefits included improved social engagement, wakefulness and a reduction of side effects related to a reduction of conventional medication dosage. The opportunity for participants to provide unstructured responses yielded novel perspectives about the program delivery in the context of normal hospital clinic service delivery. A summary of the participant characteristics is shown below in Table 1.

**Table 1:** Participant characteristics

Participant	Child Gender	Interviewee	Carer	Income	Carer Education	Diagnosis	Clinical Summary
P01	M	Mother	Mother	125+	Bachelor Degree	Lennox-Gastaut Syndrome	Initial benefit, then lost
P02	F	Mother	Mother	75-100,000	Vocational Training	AICARDI Syndrome	No difference seizures, better alertness
P03	M	Mother	Mother	25-50	Vocational Training	Lennox-Gastaut Syndrome	No difference
P04	M	Foster Father	Foster Father	75-100	High school	Lennox-Gastaut Syndrome	Better seizures and awareness
P05	M	Father	Father	Less 25	High school	Refractory epilepsy	Better seizures
P06	F	Mother	Mother	Less 25	Vocational Training	Refractory epilepsy, unknown	No benefit
P07	M	Mother	Mother	125+	Post Graduate	Refractory epilepsy, idiopathic	Better - seizures reduced
P08	M	Mother, Father	Mother	125+	Bachelor Degree	Dravet Syndrome	Nil benefit
P11	F	Mother	Mother	125+	Vocational Training	AICARDI Syndrome	No benefit from seizures - more alert
P12	M	Mother	Mother	Less 25	Vocational Training	Dravet Syndrome	Nil benefit
P13	M	Mother, Father	Mother	75-100	High school, Grade 11	Landau-Kleffner Syndrome, Pseudo-Lennox Syndrome, Continuous Spike Wave Syndrome	Side effects - no other benefit
P15	M	Father	Father	125+	Post Graduate	Dravet Syndrome	Better - seizures/alertness
P16	F	Mother	Mother	25-50	Vocational Training	Dravet Syndrome	Better
P17	F	Mother	Mother	125+	PhD	Refractory epilepsy syndrome	Better in alert - seizures ISQ
P18	F	Mother	Mother	50-75	High school	AICARDI Syndrome	Better between seizures - seizures ISQ
P19	M	Mother, Stepfather	Mother	25-50	High school	Genetic epileptic encephalopathy	Better - alert
P20	F	Mother	Mother	50-75	High school, Grade 9	Miller-Dieker Syndrome	No benefit
P21	M	Father, Mother	Mother	125+	Bachelor Degree	Dravet Syndrome	Better seizures/alertness
P22	F	Mother	Mother	100-125	Vocational Training	Refractory epilepsy, undefined	Better seizures/alertness

### Theme 1: seizure activity

Coding: refractory epilepsy, uncontrolled, ‘failure’ of other treatment modalities.

Families described their fears and concerns for their child, their disappointment with many failed treatment options, and the difficulty for their children to engage with mainstream and modified education programs. Participants explained their children had complex seizure patterns with frequent and often protracted seizure activity. Descriptions included occasions when their children experienced periods of status epilepticus, requiring hospitalisation and rescue medications as shown in Extract 1 and 2.

- **Extract 1**
*“There was one time where she was continually having seizures and the Midazolam had done nothing, so they gave her Clobazam, and she was non-responsive for quite a while”*
- **Extract 2**
*“She was having periods of hypoxia between her tonic-clonics at night. That was so scary. Basically, I felt like it was an all-night tonic-clonic, not breathing, into a tonic-clonic not breathing, into a tonic-clonic kind of cycle all night long… I was very concerned about her, because her speech started to slur, and she was just lacking in energy. She had a big change there from how she had been a few months earlier, she was full of life, to this…”*

### Theme 2: social engagement

Coding: diminished participation in family activities, diminished participation in school and learning.

Extract 3 demonstrates how seizure activity is not only disruptive for planned and structured activity such as schooling or social and community activities, it also has significant impact for the expectation of usual patterns of child growth and development, as a significant risk of hypoxia may therefore impair the expected trajectory of child and adolescent development.

- **Extract 3**
*“He would have 60+ seizures a day, would be just fatigued and really unable to do anything. You just had to put a line through your day. We would have to go back to bed and wait it out and he may or may not get kindergarten that morning.”*

Families described their desire to find any treatment option that provided some form of improvement, either in reduced seizure activity, quality of life, or improvement associated with the reduction in symptoms or side effects associated with pharmacological interventions as shown in Extract 4.

- **Extract 4:**
*“I had to resign from my work because of my son’s epilepsy and to care for him, so that I am his full-time carer. We would have done anything. At one stage we thought we would even consider going overseas to access cannabis if that was the way we had to do it. We were very aware that we couldn’t travel because he was so unstable with his epilepsy, that we wouldn’t have been travelling anyway.”*

### Theme 3: drug safety

Coding: knowledge of drug trials, legal access to Cannabidiol.

Extract 5 is an example for about a quarter of those interviewed who described accessing unregulated cannabis products prior to starting on the CAS program. The participant refers to a coastal town in New South Wales, Australia. Concerns about accessing unregulated cannabis products ranged from the drug being illegal, lack of ability to accurately identify dosing quantities and administration techniques as well as having experience of adverse side effects or a fear of unknown, adverse side effects. As shown in Extract 6, having legal access to an experimental cannabinoid product that may help their child was therefore identified as a benefit of the CAS program.

- **Extract 5:**
*“I had nipped down to “a coastal town (sic)” and gotten a little something, and it did nothing… I stopped because I was too frightened to go down and get more.”*
- **Extract 6:**
*“We were desperate for something that would work. We had no problem with the use of cannabis in a medical situation. We weren’t worried about what people thought or any stigma or anything. We had good support from people around us in (sic) approaching that sort of treatment. So really, we liked the idea that hopefully, that it was a plant-based sort of product, and felt very lucky that we had medical supervision while using it. That was a big thing for us, so before we even knew the trial was coming, we had been, sort of agitating the Epilepsy Team. We were saying: ‘is something coming? Please consider our son for that.’ We just felt it was a very great opportunity to try treatment that was a bit different from the ones that we tried and that weren’t working.”*

### Theme 4: drug efficacy

Coding: varied results, “some benefit” or “no benefit”.

Whilst families’ perceptions showed the use of CBD did not provide a therapeutic reduction in the seizure activity for all patients diagnosed with refractory epilepsy, Extract 7 and Extract 8 are examples of how it’s use as an adjunctive, safe pharmacological agent was perceived to provide other benefits by some patient’s families.

- **Extract 7:**
*“He’s been a lot better, all round I think a happier child. Sleeping better, more alert and more interactive with us as well. You can tell he’s more there.”*
- **Extract 8:**
*“She had a period of almost no seizures and then the seizures started to very gradually increase again.”*

### Theme 5: clinical support

Coding: health service provision, clinical trial administration.

Parents and caregivers’ accounts were similar regarding having no concerns about the safety of the drug being provided by the hospital pharmacy. Extract 9 illustrates how they felt they were supported through the information and explanations provided by their treating clinicians and pharmacists.

- **Extract 9:**
*“The side effects were (explained). I remember going to the appointments and getting the information about it, what to do with it, talking to the pharmacist for a couple of hours. I felt like I knew everything I needed to know and then we just got it.. To go with it and see what happens. The pharmacist was wonderful.”*

Having a dedicated registered nurse allocated to the CAS program was described as important by families as important. Extract 10 and 11 are examples of comments made by parents and caregivers. Families were contacted by the same person each time for follow up and monitoring, in this way families developed rapport and felt comfortable to provide information and data throughout the program. Participants were also able to make contact with the trial nurses when they had questions.

- **Extract 10:**
*“The clinical nurse consultant is very approachable and we’re able to access her quickly if we need to. If we have issues in terms of her script or the pharmacy prescription, they’re managed within 24 hours and always with confidence and efficiency, and we’re kept in the loop.”*
- **Extract 11:**
*“We’ve had a great experience. In regard to the doctors, her doctor who is doing it with her, the nurses that are involved in it, we haven’t had a bad experience at all and I could not complain about one person that’s been involved… Being part of a Facebook group, you see what happens outside of Australia in other countries, I am really grateful for what we have here because there are a lot of countries that are way worse off than what we are so it does give you that really good perspective of how we are treated very well.”*

After being accepted on the program, most families described their participation in the scheme as being straightforward and unproblematic. However, Extract 12 is an example of reported instances of difficulties for families travelling interstate, and not being confident about taking the CBD ‘across the border’.

- **Extract 12:**
*“If I do have an issue, it’s the lack of ability to get it from anywhere else. So when we had episodes a few months back when we had status (epilepticus), we were in the Lismore Base Hospital. We couldn’t get any supplies. We were down there for a funeral, and I wasn’t sure of the rule for transporting it over into new South Wales, so I didn’t take my Epidiolex with me, we were coming back the same day. We ended up in hospital all weekend, and couldn’t get Epidiolex. So that’s probably the only real issue I can say that I’ve had, is accessing it outside of the Queensland Children’s Hospital.”*

For some families, they felt that being part of the program meant that they had added reassurance that their child had additional clinical review time with their Neurology team. Extract 13 shows how families felt they were able to monitor their child’s overall condition more closely. Extra appointments and pathology requirements for blood tests were managed through the usual hospital booking system, and clinical service requesting pathways.

- **Extract 13:**
*“We felt that we weren’t managing a lot of his illness on our own without enough medical support. More frequent appointments was actually quite attractive to us. We wanted access to the Neurologists and if that meant that we could get more access during the trial and closer follow-up then that was a bonus for us.”*

For clinicians, combining regular planned patient clinical review meetings with CAS program reviews allowed for the program to be integrated without considerable extra clinic bookings and resourcing as shown in Extract 14.

- **Extract 14:**
*“Before we even were kind of fully accepted onto the trial and started Epidiolex, we’d had meeting with our daughter’s Neurologist who kind of took us through all the risks and potential benefits and the procedures for the trial and that kind of thing, so we were well informed before we even started her on Epidiolex.”*

### Theme 6: social acceptance of drug therapy

Coding: personal choice of family, positive generally, “whatever works”.

Families discussed the perceptions of their families and friends of participating in a trial for CBD as being generally positive. Similar reports to Extract 15 were described by participants of their families and friends adopting a ‘whatever works’ attitude for the management of refractory epilepsy.

- **Extract 15:**
*“They have all been super supportive, you get the classic jokes of: ‘can I have some’ type of thing, but they have all seen the change in her, so even if anyone was sort of ‘on the fence’ initially, they have seen the difference in her now, and they would never say it was a bad thing for her.”*

### Theme 7: program delivery

Results from the interviews with some families highlighted their determination to advocate for the CAS program and for their child’s participation. Extract 16 demonstrates the commitment expressed by many families to participating in the CAS program.

- **Extract 16:**
*“Our son’s life was on the line, we are lucky he was a fighter, or strong enough child to the point where he could trial. What about the families that don’t get that? I think refractory (epilepsy) is – you have exhausted medications, the likelihood of them not working, so how many is too many?”*

As the program had a limited number of places, the findings suggested that those families not meeting the inclusion criteria may have experienced disappointment. Extract 17 highlights the challenges of delivering a program for a novel treatment in the context of usual care health services and the perception of ‘gaps’ in the service delivery model.

- **Extract 17:**
*“I’m not sure if there was an appreciation of the emotional investment families had in attempting to access a product, and then had a lack of support if they were rejected. Maybe a better recognition by clinicians, whether that is more training, or maybe… some sort of support services for families to assist them through that process, of whether they got on it, or not or had to come off it or something like that, more of the holistic, not just the medical (support). I think that was really underestimated, just how people had been, us included, hoping that this would be the answer for years, and I don’t know that people realised. Some of the clinicians would have, maybe the clinicians involved in the trial realised just how devastating it was to some families to not be given access.”*

The CAS program was a new service model at the Queensland Children’s Hospital, evolving over the course of the CAS program delivery period. There were new processes implemented by clinicians, and at times improvements to the early model of care. Extract 18 is an example of how families contributed to the development and enhancement of the CAS program model.

- **Extract 18:**
*“We had gone in hoping that there would be really good data coming from this, about who benefitted and what happened, and things were quite disorganised. We weren’t the first, but we were maybe a bit early into it, and the paperwork and everything was quite disorganised, and the systems and the protocols were quite disorganised. I think we were a bit disappointed with that aspect. It seems the trial had started before the systems and protocols were in place, possibly, so we felt that some of the experiences that we were seeing probably weren’t documented and probably weren’t being collated across other people who were participating. We were disappointed that it was a missed opportunity”.*

## Discussion

In capturing the perceptions of family members, the authentic and often very raw narratives of families managing the everyday impacts of a chronic condition for their child were highlighted in the context of their expectations for improvement. Perspectives of parents and caregivers revealed many similarities and different experiences whilst taking part in the CBD treatment program. Key themes emerged relating participants’ expectations for the CBD treatment program to seizure activity, family and school engagement, drug safety and legal access, efficacy, clinical support, social acceptance and program delivery of the medication.

Legal access to CBD was highlighted in this study as being of importance to many parents and caregivers, illustrating how clinical trials for this patient group have addressed concerns about accessing CBD illegally, similar to findings reported in a national survey conducted in Australia [16]. The findings from this study show caregivers’ perceptions of improvement in seizure activity were observed at some point during the CAS trial for more than a third of participants. Additionally, other positive outcomes were described relating to quality of life such as improved alertness, sleep and a reduction in other anti-epileptic medication. The results are important to consider along with the findings of similar studies in New South Wales (NSW), Australia (*n* = 40), and in Israel (*n* = 74) [12, 17]. Similar to the CAS program conducted in NSW and in Israel data collection from the clinical trial included clinical efficacy, dosing titration schedules, adverse events, hospitalisations and program outcomes [12, 17]. These findings support the notion that treatment with CBD has other perceived benefits to support quality of life for participants and their families.

Whilst clinicians are familiar with the aetiology and trajectory of refractory epilepsy, it can be difficult to appreciate the lived experience of health service consumers when clinical appointments may only consist of short and infrequent interactions. Clinical case notes associated with the CAS study in NSW and a trial study conducted in Colorado, USA suggested potential for a placebo effect in the reporting of efficacy by patients’ families [12, 18]. A cross-sectional study of survey respondents using medicinal cannabis via a variety of routes suggested a high probability of selection bias leading to a high percentage of reports of cannabis efficacy in treating a wide variety of medical conditions [19]. Whilst the cohort was comprised of a small percentage of epilepsy patients (1%), the data were aggregated, and therefore efficacy for epilepsy patients was not possible [19]. Another study undertaken in Canada, aggregated data obtained from a national survey about medicinal cannabis use [20]. Whilst the survey data mentioned broad categories of health conditions, epilepsy was not mentioned, and therefore the efficacy for use in this patient group could not be ascertained [20]. The current study therefore extends the understanding not only of a dedicated CBD treatment method for a defined group of epilepsy patients, objective outcomes in addition to self-report are available for further comparison.

The findings from the current study revealed insights into caregivers and families’ social engagement in education, community and the workforce as motivation for participation in the CBD program. The personal narratives and resultant themes demonstrated how daily life was frequently disrupted by unpredictable seizure patterns associated with refractory epilepsy, and how developmental trajectories were significantly impacted. These findings are congruent with findings from an earlier review of the global burden of disease outlining the impact of social exclusion, physical risk and disrupted education and employment related to epilepsy for individuals with epilepsy and their families [6]. A report detailing the economic burden of epilepsy in Australia further supports the findings of this study by quantifying the cost of epilepsy through engagement with work and productivity in addition to overall health system costs [21]. Productivity costs are calculated to represent 19% of the total $12.3 billion annual cost of epilepsy [21]. Furthermore, the nature of the interview study provides examples to contextualise the findings of statistical studies demonstrating increased hospitalisation and mortality rates for patients diagnosed with severely drug-resistant epilepsy [7, 22].

Most participants in this study were maternal primary carers for the child diagnosed with refractory epilepsy. Many of these described working reduced hours or having to withdraw from paid work to provide full time care for their child. The qualitative data from this study therefore adds to the epidemiological evidence and work of health economists describing the negative impact of disability on family level poverty and health budgetary economic projections [23–25]. Whilst work has been done to quantify the impact of caring for a disabled family member for primary caregivers, little work has been done to describe these impacts for families caring for an individual diagnosed with refractory epilepsy [21].

In addition to the primary aim of this study, results from the interviews indicate a reliance on health service provision for caregivers supporting a person diagnosed with refractory epilepsy. Not only is the provision of a supervised and regulated open-label clinical trial of benefit to this group of patients, the additional time spent with clinicians was deemed a benefit of the CAS program for caregivers. Responses from participants in the current study suggested that long term relationships with clinicians were built up over many years of clinic interactions and hospitalisations. DeRigne describes a review of literature for the financial and care concepts relating to the model of a ‘medical home’ to provide holistic patient-centred care for families supporting a child with disability [26]. Findings were summarised in three broad domains of family out-of-pocket expenses, impact on family employment and the role of the medical home in moderating these effects [26]. The success of such a model relied on meeting the needs of the individual, having a health advocate who understood the system and the patients’ needs and appropriate use of resources to meet the needs of the patients [26].

This study suggests that the current health service provision for this group of patients relies heavily on the commitment and capacity of the family members supporting them and that more work is required to meet the needs of patients diagnosed with refractory epilepsy and their families. Enhancing existing health service delivery options may include addressing the perceptions of families that they require additional specialist supervision. These needs may be met through existing community-based services provided through general medical practice. Additional training for doctors and health care providers for families with special health care needs may help to address these needs.

The extension of the clinical service delivery model to include clinical trials allowed for close monitoring and evaluation of the CBD program to improve and enhance processes associated with other programs of care. Lessons learned during the program led to the allocation of a dedicated registered nurse for family contact and communications. Accessibility options for patients to participate in novel therapies are subject to inclusion criteria and limited numbers of participants, differing from usual care models in which clinical decision making is based on symptom management with approved therapies. Future enhancements were suggested to meet the psychosocial needs of families not eligible to participate in studies or families of patients who withdrew from the program as a result of adverse events or lack of symptom improvement associated with the novel therapy.

Several strengths can be identified for this study, firstly, undertaking an interview study provides subjective perceptions and insights of families that can be compared to objective clinical data, illuminating the motivations for families to participate in clinical trials, potential for reporting bias based on families’ expectations for treatment with CBD and other factors associated with the use of a novel experimental therapy. Secondly, the CCTRND extended the health service experience many patients were already engaging with by providing clinical trials for this patient group. Thirdly, the findings of an interview study have allowed for the perceptions of families to be explored more fully to understand how benefits of treatment for patients may include factors other than improved seizure activity. For the patient and their family, the management of a chronic condition over many years may involve many primary health care providers, in addition to education and community services. Having an understanding of how their child’s condition may improve in other ways helps to establish the usefulness of CBD as an adjunctive therapy.

Along with the strengths of this study, limitations should also be mentioned. Whilst participation in this study was available to all patients in the QLD CAS program, not all families were interviewed due to time and resource constraints. Therefore, this study is not a reflection of the perceptions of all families involved in the program, nor is it possible to state that the perceptions and experiences are similar to families involved in other CAS programs provided by other health care facilities. The potential for a placebo effect has been identified for this open label study. The ability to compare the findings from this study with objective electroencephalography (EEG) data will serve to reduce, but not eliminate all aspects of potential placebo effect. Finally, the severity and heterogeneity of patients’ diagnoses, multi-pharmacological management of their epilepsy and lack of drug assessment criteria were all factors with potential to influence patients experiences during the CAS program.

While the CAS program is currently closed for new participant families in Queensland, the review of clinical trial data for CBD as a novel treatment is ongoing in Australia. The findings from this study can be used for comparison with objective data for this patient group. Results of clinical trials done here, coincide with clinical trial studies being undertaken around the world, the findings from which are fundamental for the provision of safe therapeutic drugs for our patient populations, and are part of the evidence-based pathway required for systematic global regulatory reform for this class of drugs. The program here in Queensland has demonstrated the ability of the Children’s Health Queensland Hospital and Health System to respond to consumer health needs for dispensing an experimental therapy in a regulated manner, not only supporting Queensland families, but also contributing to the advancement of health science research internationally.

## Conclusion

This study provided unique perspectives of families’ experiences managing untreatable epilepsy, their experiences with conventional and experimental pharmacological treatments and health services. Whilst families’ perceptions showed the use of CBD did not provide a therapeutic reduction in the seizure activity for all patients diagnosed with refractory epilepsy, the use of CBD as an additional, pharmacological agent was perceived to provide other benefits by some patient families. Future clinical trials of novel treatments may need to consider the assistance provided to families of children in addition to seizure control, for those who were not eligible for trialling new therapies, or who withdrew from trials with adverse side effects. Further research is warranted to compare the findings from this study with electroencephalographic data collected for patients enrolled in the *Compassionate Access Scheme*. By combining qualitative and quantitative data for this patient population, greater insight into the efficacy of Cannabidiol may be possible.

## Data Availability

Whilst the datasets generated and analysed during the current study are not publicly available due to ethics restrictions applicable to privacy in relation to identifiable information of children, they are available from the corresponding author on reasonable request.

## Abbreviations

AED: Anti-epileptic drug
CAS: Compassionate Access Scheme
CBD: Cannabidiol
CCTRND: Centre for Clinical Trials in Rare Neurodevelopmental Disorders
DALY: Disability adjusted life year
GCP: Good clinical practice
NSW: New South Wales
QCH: Queensland Children’s Hospital
YLD: Years of life lived with disability
YLL: Years of life lost
